# Lactate metabolism and lactylation in uveal melanoma: from metabolic adaptation to immune regulation

**DOI:** 10.3389/fimmu.2026.1965851

**Published:** 2026-09-28

**Authors:** Yingluan Wang, Zhan Cao, Dawei Sun, Lijun Qu

**Affiliations:** 1 Department of Ophthalmology, The Second Affiliated Hospital of Harbin Medical University, Harbin, China; 2 Department of Neurology, The Second Affiliated Hospital of Harbin Medical University, Harbin, China

**Keywords:** immune escape, lactate, lactylation, metabolic adaptation, quiescence-like state, RNA modification, tumor–immune metabolic communication, uveal melanoma

## Abstract

Uveal melanoma (UM) is the most common primary intraocular malignancy in adults. The liver is its most common site of metastasis, and in some patients, distant metastases may become clinically apparent years after treatment of the primary tumor. Metastatic UM generally shows limited responsiveness to immune checkpoint blockade. Although genetic alterations in GNAQ, GNA11, BAP1, SF3B1, and EIF1AX are important for the molecular classification and prognostic stratification of UM, these factors do not fully explain late metastatic recurrence, persistence of low-proliferative tumor cell states, immune escape, or adaptation to the hepatic metastatic microenvironment. Lactate is not only a major product of glycolysis but also an immunometabolic regulator. Through monocarboxylate transporter-mediated transport, HCAR1/GPR81 signaling, extracellular vesicle-associated transfer, and lysine lactylation, lactate influences tumor cell states, tumor–immune metabolic communication, chromatin regulation, and post-transcriptional control. In UM cell studies, exogenous lactate induces a quiescence-like phenotype characterized by reduced proliferation and migration, increased oxidative metabolism, changes in chromatin organization, and elevated H3K18la levels. Tumor-derived lactate increases the stability of PD-L1 and HLA-E proteins through GPR81-related signaling, enhancing the resistance of UM cells to T cell- and NK cell-mediated killing. Under hypoxic conditions, extracellular vesicle-associated lactate participates in metabolic communication between tumor cells and immune cells, alters macrophage states, and impairs CD8^+^ T-cell effector function. Lactylation further links lactate metabolism with chromatin regulation and RNA modification-related post-transcriptional control. Studies in related ocular melanoma models indicate that H3K18la regulates YTHDF2 and ALKBH3 and thereby affects m^6^A- and m¹A-associated RNA regulation. This review summarizes current studies on lactate production, transport, receptor signaling, extracellular vesicle-associated lactate transfer, and lactylation in UM and related ocular melanoma models, and discusses their roles in low-proliferative adaptation, tumor–immune metabolic communication, hepatic metastatic microenvironment adaptation, and therapeutic resistance.

## 1 Introduction

Uveal melanoma (UM) is the most common primary intraocular malignancy in adults (1). Local treatments such as radiotherapy, local resection, and enucleation can control most primary tumors, but distant metastasis remains the leading factor shaping patient prognosis, with the liver being the most common metastatic site (2). In some patients, metastases appear years after treatment of the primary tumor, suggesting that disseminated UM cells may survive for prolonged periods without clinical detection and resume proliferation under favorable conditions. Compared with cutaneous melanoma, UM shows limited overall responses to conventional chemotherapy and immune checkpoint inhibitors. Therapeutic resistance in UM is associated with low tumor mutational burden, ocular immune privilege, impaired antigen presentation, and hepatic immune tolerance. It may also be influenced by the combined effects of metabolic adaptation and epigenetic dysregulation.

Current research on UM development and progression has primarily focused on key genetic alterations involving GNAQ, GNA11, BAP1, SF3B1 (3), and EIF1AX, as well as chromosomal abnormalities, molecular prognostic classification, liver metastasis mechanisms, immunotherapy, metabolic reprogramming, and classical epigenetic regulation (4–7). These studies have provided an important foundation for understanding UM molecular subtypes, metastatic risk, and therapeutic resistance. However, how altered lactate metabolism and lactylation contribute to cellular state transitions, immune escape, and adaptation to the liver metastatic microenvironment in UM remains insufficiently integrated. In particular, the role of lactate as a metabolic regulator of tumor cell states, immune regulation, and hepatic metastatic adaptation has not been systematically summarized in UM. In the setting of late metastatic recurrence, limited responsiveness to immune checkpoint blockade, and hepatic metastatic adaptation, lactate may have roles beyond being a byproduct of enhanced glycolysis. Lactate-related processes are particularly relevant to UM because they are linked to low-proliferative tumor cell states, tumor–immune metabolic communication, immune escape, and the liver metastatic microenvironment.

Lactate research has moved beyond the view of lactate as only a glycolytic end product. Lactate is now discussed as a metabolic substrate, a regulator of the local microenvironment, a ligand for receptor-mediated signaling, and a source of lactyl groups for protein lactylation (8). The discovery of lysine lactylation (Kla) further showed that lactate-associated metabolic states can affect chromatin regulation, protein function, and post-transcriptional processes through histone and non-histone protein modifications (9). These functions allow lactate and lactate-associated lactylation to be considered in relation to UM cell-state regulation, immune microenvironment remodeling, and metastatic adaptation.

Clinical studies have reported higher L-lactate levels in the aqueous humor of patients with UM, with a possible association with tumor proliferative activity (10). *In vitro* work has shown that exogenous lactate can induce a quiescence-like phenotype in UM cells, accompanied by changes in metabolic state and chromatin structure (11). In the immune setting, UM cells can stabilize programmed death-ligand 1 (PD-L1) and human leukocyte antigen E (HLA-E) through interleukin-6 (IL-6)-driven autocrine lactate signaling (12). Under hypoxia, lactate carried by extracellular vesicles (EVs) may also participate in immune cell reprogramming (13). Studies in ocular melanoma further found that histone H3 lysine 18 lactylation (H3K18la) can regulate RNA modification-related factors, including YTHDF2 and ALKBH3, and affect the stability, degradation, and translation efficiency of specific transcripts. These studies connect lactate with UM cell state, immune regulation, and post-transcriptional control, and also suggest that lactylation may participate in the effects of lactate-associated metabolic states on chromatin and RNA regulatory processes (14, 15). Because UM differs from other ocular melanomas and other solid tumors in genetic background, metastatic pattern, and treatment response, this review distinguishes among UM studies, related ocular melanoma studies, and non-UM tumor studies. The review remains centered on UM. Studies of other ocular melanomas are included when they address lactylation mechanisms that have not been examined separately in UM.

This review examines lactate-related regulation in UM at two connected levels. The first includes lactate production, transport, HCAR1/GPR81 signaling, and extracellular vesicle-associated transfer, which determine how lactate is generated, exchanged, and sensed by tumor and immune cells. The second includes histone and non-histone lactylation, through which lactate-associated metabolic states may affect chromatin regulation, RNA regulation, and protein function. These processes are discussed in relation to low-proliferative adaptation, immune escape, and the hepatic metastatic microenvironment, followed by their therapeutic implications. **Figure 1** summarizes these relationships. Its H3K18la-associated RNA-regulatory branch is shown in greater detail in **Figure 2**. Lactylation is considered as one component of lactate-related regulation rather than an established central determinant of immunotherapy response in UM.

**Figure 1:**
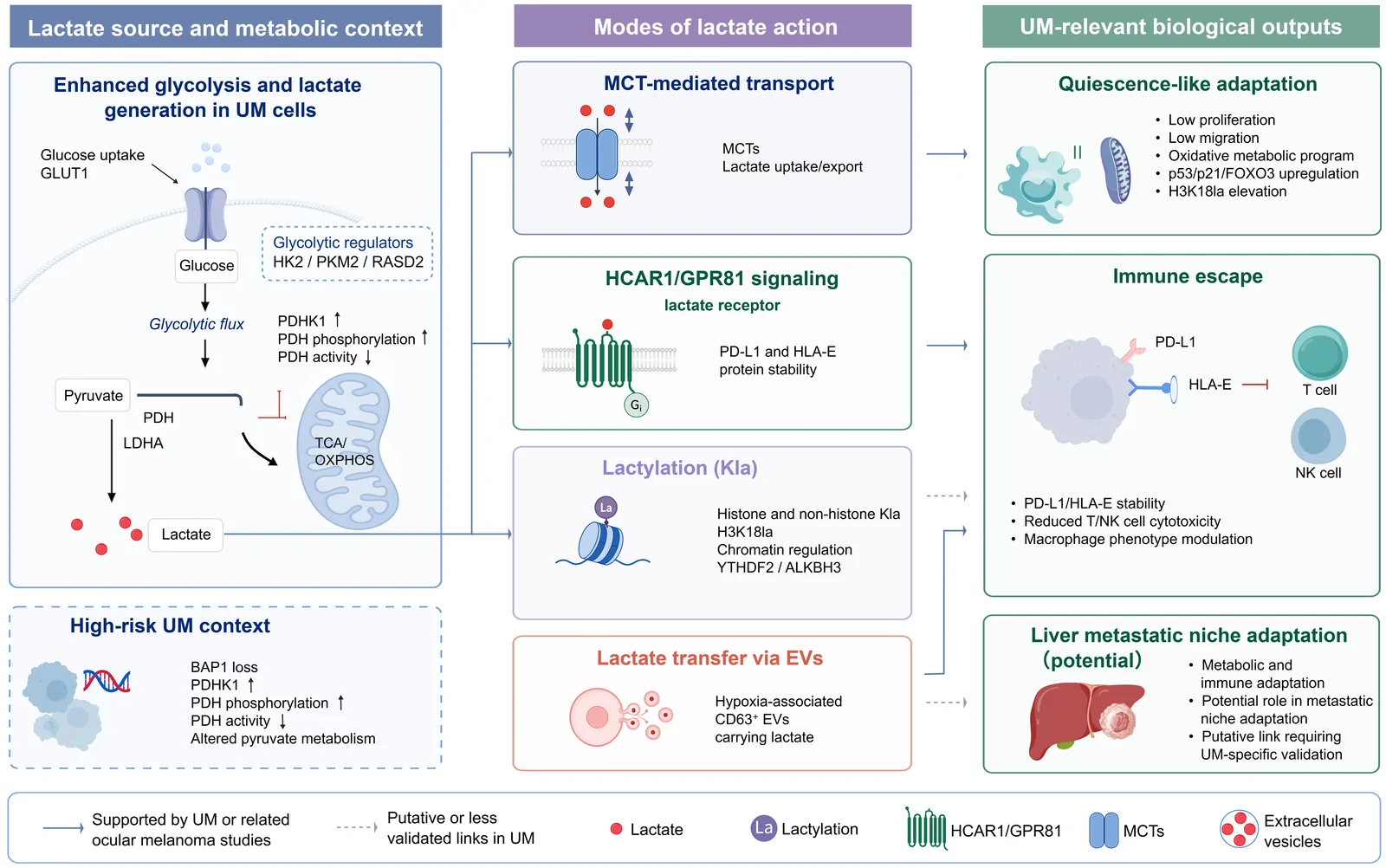
Overall framework of lactate metabolism and lactylation in uveal melanoma. Enhanced glycolysis may increase lactate production in UM cells. Lactate may participate in UM cell state regulation and tumor microenvironment remodeling through MCT-mediated uptake or export, HCAR1/GPR81 receptor signaling, lysine lactylation, and extracellular vesicle-mediated lactate transfer. These processes may be associated with quiescence-like adaptation, immune escape, and adaptation to the liver metastatic microenvironment. The H3K18la-YTHDF2/ALKBH3 RNA-regulatory branch is detailed in **Figure 2**. Solid arrows indicate relationships supported by existing studies in UM or related ocular melanoma models, whereas dashed arrows indicate relationships that remain insufficiently characterized in UM.

**Figure 2:**
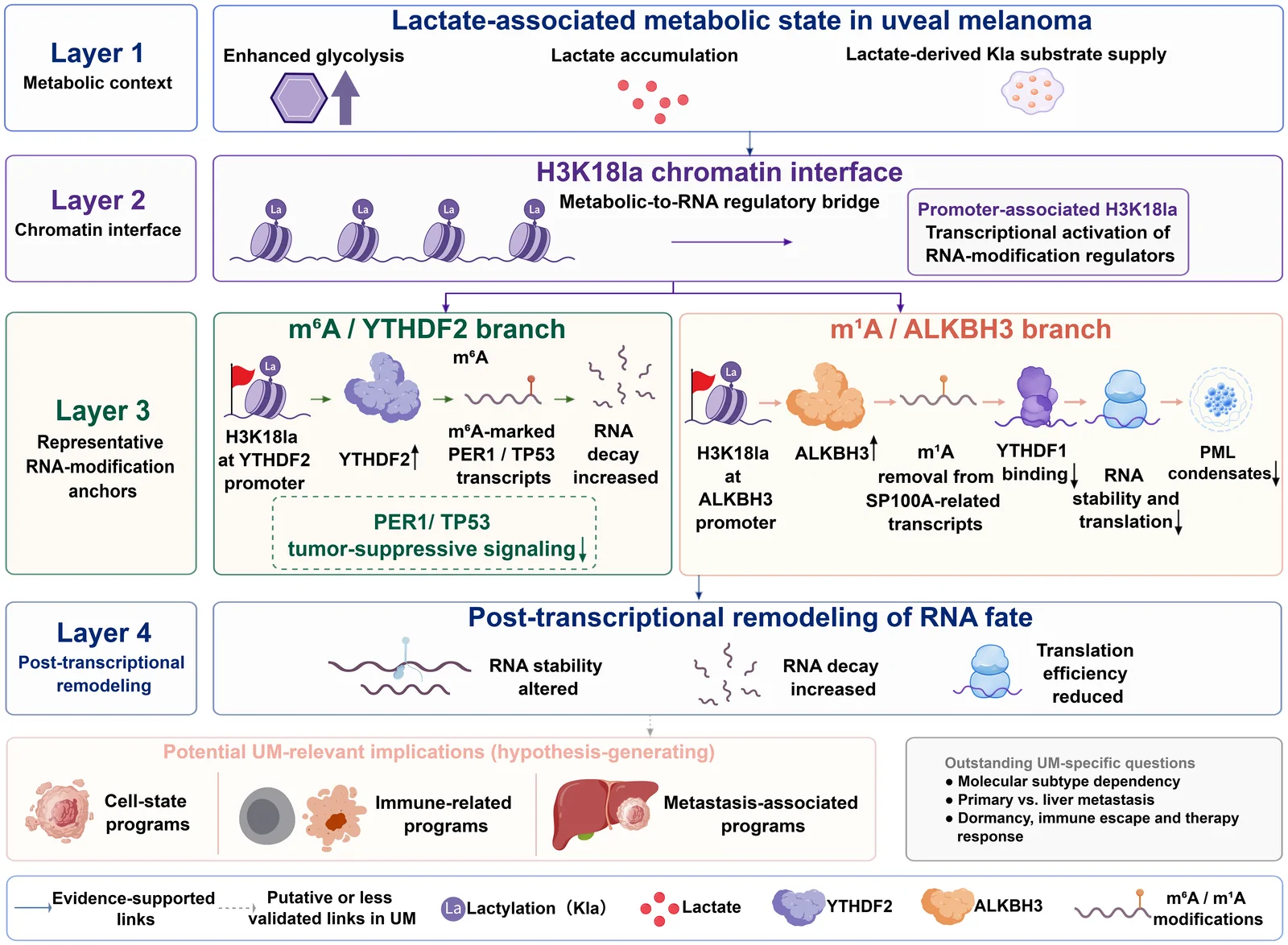
H3K18la-associated regulation of m^6^A- and m¹A-related RNA processes in ocular melanoma. Lactate-associated metabolic states may be accompanied by increased H3K18la levels and regulate the expression of RNA modification-related factors, including YTHDF2 and ALKBH3, through H3K18la enrichment at promoter regions. These changes may subsequently influence the stability, degradation, and translation of m^6^A- or m¹A-modified transcripts. The YTHDF2 and ALKBH3 branches summarize the relatively well-characterized H3K18la-associated molecular relationships identified in current ocular melanoma studies. Their roles in UM cell state regulation, immune responses, and metastatic processes remain insufficiently characterized. Solid lines indicate relationships supported by existing studies, whereas dashed lines indicate candidate relationships that require further investigation in UM. The accessibility of lactate-associated Kla substrates shown in the figure represents a conceptual illustration and does not indicate the presence of specific lactyl-group donor pathways that have been established in UM.

## 2 Molecular basis of lactate metabolism and lactylation

During enhanced glycolysis, lactate is generated as one of the main metabolic products. After increased glucose uptake, tumor cells convert pyruvate to lactate through lactate dehydrogenase A (LDHA), while regenerating NAD^+^ to sustain glycolytic activity (16). Lactate can then cross the cell membrane through monocarboxylate transporters (MCTs), allowing metabolic exchange among tumor cells, stromal cells, and immune cells (17). This transport process is also relevant to immune metabolism, since activated lymphocytes require lactate handling to maintain glycolytic activity and effector function. Extracellular lactate may activate hydroxycarboxylic acid receptor 1 (HCAR1/GPR81) and thereby regulate cellular metabolism and signaling. In this way, lactate acts not only as a metabolic product and energy substrate, but also as a signaling molecule in intercellular communication and as a metabolic precursor for protein lactylation (18).

The identification of lysine lactylation (Kla) connected lactate metabolism with protein post-translational modification and gene regulation (19). In this modification, a lactyl group is added to the ϵ-amino group of lysine residues. Zhang et al. identified histone lactylation at several core histone sites, including H3K18, H3K23, H4K8, and H4K12 (20). Their study also supported lactate as a source of lactyl groups involved in chromatin-associated transcriptional regulation. In macrophage models, lactate accumulation was accompanied by increased histone lactylation and higher expression of homeostatic and tissue repair-related genes, including Arg1. Glycolysis-derived lactate can therefore affect chromatin regulation through histone modification and link metabolic changes with specific transcriptional responses. Because of its promoter enrichment and relatively extensive functional characterization, H3K18la has become one of the better-studied histone lactylation sites.

Lysine lactylation is not a single, chemically uniform modification, but includes several chemically distinct forms. Recent biochemical studies have distinguished lysine L-lactylation from related isomeric or structurally similar modifications, including D-lactylation and N-ϵ-carboxyethyl-lysine (21). Glycolysis-associated histone lactylation is mainly composed of lysine L-lactylation, whereas other lactylation-like modifications may arise through different metabolic routes. When pan-Kla or site-specific antibody signals are used to assess lactylation changes, it is therefore important to consider whether these signals correspond to specific lactate-derived modifications.

Lactylation is not restricted to histones. Lactylome profiling has shown that many non-histone proteins in the nucleus, cytoplasm, and mitochondria can also undergo Kla modification. These proteins are involved in processes such as glycolysis, the tricarboxylic acid cycle, oxidative phosphorylation, RNA metabolism, protein homeostasis, and signal transduction. Histone lactylation mainly acts through changes in chromatin state and transcriptional regulation, whereas non-histone lactylation can directly alter substrate protein stability, enzymatic activity, subcellular localization, and molecular interactions. Xiao et al. further noted that lactate can form N-lactoyl amino acids with free amino acids in addition to contributing to protein lysine lactylation, suggesting that lactate-associated modifications are not limited to Kla (9). These findings broaden the role of lactate in cellular regulation, although the functional significance of these modifications in tumors remains to be clarified.

Lactylation is a dynamic process regulated by enzymatic activities. Lysine acyltransferases such as p300 may participate in certain histone lactylation processes (20). Recent studies have further shown that ACSS2 can function as a lactyl-CoA synthetase to convert lactate into lactyl-CoA and form a functional coupling with KAT2A, thereby promoting histone H3 lactylation and related transcriptional programs (22). During the delactylation process, HDAC1–3 exhibit strong histone delactylase activity (23). SIRT1 and SIRT3 can also remove Kla under specific cellular and substrate contexts. SIRT1 has been associated with the regulation of H3K18la, whereas SIRT3 can act on H4K16la and certain non-histone substrates related to mitochondrial function or cell cycle regulation (24).

## 3 Lactate metabolic alterations in UM

Metabolic reprogramming in UM spans glycolysis, oxidative phosphorylation, lipid metabolism, amino acid metabolism, and other pathways, and varies with genetic background, cell state, and microenvironmental conditions (4). Lactate lies between glycolytic and oxidative metabolism. In UM, its biological relevance is shaped not only by how much lactate is produced, but also by how it is transported across membranes, used inside cells, transmitted between cells, and sensed through receptor-mediated signaling. For this reason, lactate metabolism in UM needs to be considered at several levels, including glycolysis-associated lactate production, intraocular lactate accumulation, and lactate uptake and utilization by tumor cells.

Multiple glycolysis-related regulatory pathways can influence lactate production in UM. The miR-216a-5p/HK2 axis has been reported to regulate glycolysis, lactate production, and tumor growth in UM cells through modulation of the Warburg effect, suggesting that hexokinase 2 (HK2)-mediated glucose metabolism contributes to malignant phenotypes in UM (25). RASD2 has also been reported to upregulate glycolysis-related molecules, including GLUT1, HK2, PKM2, and LDHA, and promote growth, migration, and metastasis-related phenotypes of UM cells (26). These studies indicate a functional association between glycolytic reprogramming and UM progression and provide a metabolic basis for increased lactate production. However, findings related to HK2 and RASD2 mainly reflect alterations in glycolytic pathways, and whether these changes result in sustained lactate accumulation also depends on lactate transport, oxidative utilization, and lactate uptake and utilization by microenvironmental cells.

Metabolic measurements in clinical samples suggest the presence of altered lactate metabolism in the local ocular environment of UM. Recent studies have shown that L-lactate levels in the aqueous humor of patients with UM are higher than those in cataract controls and are associated with pathological mitotic activity (10). These findings suggest that aqueous humor lactate may reflect metabolic alterations in the ocular microenvironment of UM and may be associated with tumor proliferative activity. Available studies have not shown consistent associations between aqueous humor lactate levels and tumor size, major histological subtypes, gene expression classes, or metastatic status. Therefore, aqueous humor lactate is currently more suitable as an indicator of local metabolic alterations rather than a direct marker for molecular classification or metastatic risk assessment. Some clinical studies have also reported that serum lactate dehydrogenase (LDH) levels are associated with overall survival in patients with metastatic UM (27). Unlike aqueous humor lactate, serum LDH primarily reflects systemic tumor burden, tissue injury, and whole-body metabolic status and cannot be used as a surrogate marker for local tumor lactate levels or lactate utilization status.

Exogenous lactate reduced proliferation and migration in UM cells, accompanied by increased oxidative metabolism and H3K18la levels (11). MCT1 inhibition attenuated this response, whereas HCAR1 activation alone did not reproduce it, pointing to a role for lactate uptake and intracellular utilization.

Autocrine lactate generated during sustained IL-6 stimulation stabilized PD-L1 and HLA-E through GPR81 signaling and increased UM cell resistance to T cell- and NK cell-mediated cytotoxicity (12). Lactate can therefore affect UM cells through metabolic utilization and receptor-mediated signaling. The outcome varies with lactate source, duration of exposure, transport route, and the metabolic state of the responding cells. The next two sections examine how lactylation affects chromatin and RNA regulation in ocular melanoma and how these findings relate to UM.

## 4 H3K18la in ocular melanoma and its relevance to UM

High-risk UM progression often involves both chromatin remodeling and changes in cellular metabolism. Integrated genomic studies have defined molecular subgroups of UM and linked monosomy 3 and BAP1 loss to poor-prognosis tumors with characteristic methylation and transcriptional profiles (28). BAP1 deficiency can disrupt melanocytic differentiation and favor stemness-related phenotypes in UM cells (29). Histone modification profiling has also shown that UM tissues differ from normal uveal melanocytes across several post-translational histone marks, with selected acetylation sites and H4K20me1 associated with BAP1 status (30). Metabolic studies add another layer of heterogeneity. Some BAP1-mutant UM subtypes show increased glycolysis and nucleotide biosynthesis, whereas others rely more heavily on fatty acid oxidation (31). Increased pyruvate dehydrogenase kinase 1 expression and PDH phosphorylation further point to altered pyruvate metabolism in a subset of BAP1-mutant UM (32). In high-risk UM, chromatin remodeling and metabolic adaptation may therefore occur side by side, making this setting relevant for studies of lactylation-related regulation.

H3K18la is among the better-studied lactylation sites in ocular melanoma. Studies using ocular melanoma tissues and cell models have reported higher global lysine lactylation and increased H3K18la levels, with H3K18la abundance associated with recurrence risk and clinical outcome (14). Chromatin immunoprecipitation sequencing further showed that H3K18la is enriched mainly at gene promoters and overlaps substantially with transcriptionally active regions. These findings suggest that H3K18la is not only associated with lactate-related metabolic states but also exhibits promoter enrichment patterns and is linked to specific transcriptional regulatory events.

Further studies suggest that the functional roles of H3K18la may involve interactions between chromatin regulation and post-transcriptional regulatory processes. H3K18la is enriched at the promoter region of YTHDF2 and is associated with YTHDF2 transcriptional activation (14). In addition, H3K18la has been found to be associated with the regulation of ALKBH3 expression (15). YTHDF2 and ALKBH3 are involved in the regulation of m^6^A and N1-methyladenosine (m¹A) RNA modifications, respectively. Changes in the expression of these factors suggest that lactylation may influence RNA modification regulatory networks. Therefore, H3K18la may not only represent an epigenetic response to lactate metabolic states but may also participate in linking chromatin regulation with post-transcriptional regulatory processes.

The H3K18la studies summarized here used ocular melanoma tissue cohorts together with UM-derived and conjunctival melanoma cell lines. The YTHDF2 study included UM-derived cell lines such as 92.1, MUM2B, and OCM1, as well as conjunctival melanoma cell lines including CRMM1 and CRMM2 (14). The ALKBH3 study also used both UM and conjunctival melanoma cell lines (15). Because UM and conjunctival melanoma differ in molecular background, metastatic pattern, and treatment response, these mechanisms are discussed in the context of ocular melanoma rather than presented as UM-specific mechanisms. Although UM-derived cell lines were included, these relationships have not been examined across molecularly characterized primary UM tissues, UM molecular subtypes, or UM liver metastases.

## 5 Lactylation-related RNA regulation in ocular melanoma and its relevance to UM

YTHDF1 recognizes m^6^A-modified HINT2 transcripts and promotes their recruitment to polysomes, which increases HINT2 protein expression and suppresses tumor cell proliferation (33). In UM cells and xenograft models, higher ALKBH5 expression has been associated with poorer prognosis. Reducing ALKBH5 expression suppresses UM cell proliferation, migration, and invasion and limits tumor growth *in vivo* (34). Mechanistically, EP300-mediated H3K27 acetylation promotes ALKBH5 transcription. Increased ALKBH5 then removes m^6^A modifications from FOXM1 transcripts, increases FOXM1 mRNA stability, and promotes UM progression (34). TCGA and GEO dataset analyses further indicate that the expression patterns of m^6^A regulatory factors are associated with chromosome 3 status, molecular subtypes, and patient survival (35). These studies suggest that RNA modifications do not function as unidirectional regulators in UM, and their effects depend on the type of modification, the regulatory proteins involved, and the biological functions of their target transcripts.

Studies on H3K18la-mediated regulation of YTHDF2 expression have revealed a potential connection between lactylation and m^6^A-mediated RNA degradation. In ocular melanoma cells, lactate accumulation is accompanied by increased H3K18la levels, and H3K18la is enriched at the YTHDF2 promoter region and associated with enhanced transcriptional activity of YTHDF2 (14). As an m^6^A reader, YTHDF2 recognizes m^6^A-modified PER1 and TP53 transcripts, promotes their degradation, and reduces related tumor-suppressive signaling. In functional experiments, YTHDF2 overexpression partially restored the loss of proliferative and migratory capacity after lactylation inhibition, indicating that YTHDF2 may act downstream of H3K18la-associated tumor phenotypic changes. In this setting, changes in lactate metabolism may affect m^6^A reader expression through H3K18la and thereby alter the stability of specific transcripts.

The regulation of ALKBH3 by H3K18la further expands the connection between lactylation and m¹A modification. In high-risk ocular melanoma, increased ALKBH3 expression is accompanied by reduced global m¹A levels (15). Studies have shown that H3K18la is enriched at the ALKBH3 promoter region and promotes its transcription. Increased ALKBH3 levels reduce m¹A modification on SP100A-related transcript isoforms, decrease YTHDF1 binding to these transcripts, and impair RNA stability and translation efficiency, resulting in reduced expression of SP100A-related proteins and decreased PML nuclear condensates. PML nuclear condensates are involved in transcriptional regulation, protein homeostasis, and tumor suppressive responses. Their reduction suggests that lactylation-associated transcriptional regulation may further influence nuclear structures and tumor suppressive programs through RNA modification processes.

In ocular melanoma studies, promoter-associated H3K18la increases the expression of YTHDF2 or ALKBH3, followed by changes in m^6^A-dependent transcript degradation or m¹A-associated transcript stability and translation, respectively. The different effects of YTHDF1, YTHDF2, ALKBH5, and ALKBH3 show that the biological consequences of RNA modification depend on the regulatory protein and target transcript involved.

**Figure 2** expands the H3K18la-associated RNA-regulatory branch shown in **Figure 1**. It summarizes the H3K18la-YTHDF2-m^6^A-PER1/TP53 and H3K18la-ALKBH3-m¹A-SP100A relationships. Although these studies included UM-derived cell lines, they also included conjunctival melanoma cell lines, and the two relationships have not been examined in molecularly characterized primary UM tissues or UM liver metastases. Accordingly, they are discussed in the context of ocular melanoma rather than presented as established UM-specific mechanisms. Beyond RNA regulation, lactate also alters the proliferative state of UM cells, as discussed in the following section.

## 6 Lactate-induced quiescence-like state in UM

Quiescence and tumor dormancy are related but distinct biological concepts. Quiescence mainly refers to the reversible exit of individual cells from the proliferative cycle, which is typically characterized by G0/G1 cell-cycle arrest, reduced proliferation markers, and increased expression of cell-cycle inhibitors. Tumor dormancy is an *in vivo* phenomenon in which disseminated tumor cells or micrometastatic lesions persist for long periods without becoming clinically detectable. This state can be shaped by intrinsic cellular quiescence, limited angiogenesis, immune surveillance, and the microenvironment of distant organs (36). Quiescence should also be distinguished from cellular senescence. Quiescent cells retain the ability to re-enter the cell cycle, whereas senescent cells generally exhibit more stable proliferative arrest and may develop a specific secretory phenotype. Therefore, reduced proliferation observed in *in vitro* experiments should not be directly interpreted as tumor dormancy *in vivo*, but rather as a quiescence-like state or a low-proliferative state. Whether this state has implications for delayed metastasis depends on whether cells can survive long-term under low-proliferative conditions and re-enter the proliferative cycle following changes in the surrounding environment.

The clinical course of delayed metastasis in UM suggests that low-proliferative states may contribute to the long-term persistence of disseminated tumor cells. After local control of the primary tumor, some patients still develop distant metastases years or even decades later, indicating that tumor cell dissemination may occur before clinical diagnosis (1). Gill et al. analyzed postmortem tissues and found UM micrometastases in all five patients with detectable metastatic lesions and in five of six patients without clinically apparent metastases (36). These micrometastases were detected in multiple organs, including the lungs, kidneys, myocardium, and bone marrow, with the highest density observed in liver tissues. They were generally accompanied by immune cell infiltration and very low or absent proliferative activity. The postmortem observations show that disseminated UM cells can persist with little or no proliferative activity, whereas Longhitano et al. showed that lactate exposure can induce a low-proliferative phenotype in UM cells *in vitro*. The shared feature is reduced proliferative activity; the *in vitro* experiments do not reproduce the angiogenic, immune, and organ-specific components of tumor dormancy. The role of lactate in maintaining dormant UM micrometastases *in vivo* has not been directly examined. The relevance of the *in vitro* phenotype therefore depends on whether disseminated UM cells encounter high lactate levels and retain the capacity to resume proliferation.

The lactate-induced quiescence-like phenotype in UM has been mainly characterized in the study by Longhitano et al. In 92.1 UM cells, treatment with 20 mmol/L lactate reduced cell proliferation and migration capacity and was accompanied by increased expression of SIRT1, PGC-1α, TFAM, and multiple respiratory chain-related genes (11). MCT1 inhibition partially attenuated the anti-proliferative effects of lactate, whereas selective activation of HCAR1 alone did not induce similar cellular state changes, suggesting that lactate uptake and intracellular utilization are important components of this process. Lactate treatment also resulted in reduced heterochromatin proportion, increased nuclear size, elevated H3K18la levels, and increased expression of p53, p21, and FOXO3. The parallel changes in metabolic gene expression, chromatin structure, and cell-cycle regulatory molecules indicate that lactate-induced low proliferation is not just growth inhibition. It more closely resembles a quiescence-like phenotype with increased oxidative metabolism and chromatin remodeling.

This phenotype may be described as a lactate-induced quiescence-like state, characterized by reduced proliferation and migration, enhanced oxidative metabolism-related programs, increased expression of cell-cycle inhibitory molecules, and altered chromatin states. p53, p21, and FOXO3 can participate in quiescence maintenance, but they may also be involved in stress responses or senescence-related processes. Therefore, reduced proliferation alone is insufficient to determine whether this phenotype represents reversible quiescence. Future studies should investigate whether cells can re-enter the cell cycle after lactate withdrawal and whether they retain long-term clonogenic capacity, in order to distinguish reversible quiescence-like adaptation from irreversible proliferative arrest.

The concurrent increase in H3K18la and chromatin relaxation raises the possibility that lactylation contributes to gene-expression changes in this low-proliferative state. Its role in maintaining this state can be examined through cell-cycle re-entry and clonogenic regrowth after lactate withdrawal.

Overall, lactate-induced quiescence-like states provide a useful cellular system for examining low-proliferative adaptation in UM. Under high-lactate conditions, UM cells may adjust oxidative metabolism and chromatin states through lactate uptake and intracellular metabolic utilization, accompanied by reduced proliferative and migratory activities. H3K18la may participate in part of the associated gene-expression changes. Accordingly, lactate-induced quiescence-like states should be regarded as an *in vitro* low-proliferative cellular state whose relevance to delayed metastatic recurrence depends on whether comparable viable and reactivatable states occur in patient-derived and liver-associated systems.

## 7 Lactate-associated immunosuppression and the potential role of lactylation

The overall response of metastatic UM to immune checkpoint inhibitors remains limited. A meta-analysis including 36 studies, 41 cohorts, and 1, 414 patients showed that the pooled objective response rate of immune checkpoint inhibitor therapy in metastatic UM was 5.6%. Dual checkpoint blockade demonstrated higher efficacy than monotherapy, but the objective response rate remained only 12.4% (37). By contrast, tebentafusp, a gp100-directed T-cell receptor bispecific molecule, has improved overall survival in HLA-A*02:01-positive metastatic UM (38). This distinction indicates that limited responses to checkpoint blockade do not exclude T-cell-directed therapeutic activity in UM. The influence of lactate-rich hepatic metastatic microenvironments on tebentafusp-driven T-cell recruitment, activation, or persistence has not been defined. Low tumor mutational burden, ocular immune privilege, impaired antigen presentation, and hepatic immune tolerance may collectively limit the immunogenicity and effective immune responses of UM. Beyond these classical factors, lactate-related metabolic alterations within metastatic lesions may affect both UM cells and immune cells. In UM cells, lactate may increase PD-L1 and HLA-E protein stability, whereas lactate-rich conditions may impair T-cell and NK-cell effector function and alter macrophage phenotypes.

Lactate can directly suppress effector lymphocyte function at the metabolic level. Activated Activated CD8^+^ T cells depend on glycolysis for proliferation, cytokine production, and cytotoxic activity. When extracellular lactate levels are high and the surrounding environment becomes more acidic, the transmembrane lactate gradient can be disturbed, limiting MCT1-mediated lactate export from T cells and impairing glycolytic adaptation and effector function (39). Tumor cell-derived lactate produced through LDHA can also reduce IFN-γ production and cytotoxic activity in CD8^+^ T cells and NK cells (40). Lactate also acts on myeloid cells. In macrophages, tumor-derived lactate may induce ARG1 and VEGFA expression through HIF-1α-associated mechanisms (41), favoring a tumor-supportive phenotype and, in some tumor models, promoting MDSC accumulation and their suppressive effects on NK cells (42). Thus, lactate-associated immunosuppression is not simply a consequence of low pH. It reflects the combined effects of lactate concentration, proton load, transmembrane transport, and cell type-specific metabolic responses.

Previous studies have shown that lactate may enhance intrinsic immune resistance of UM cells through GPR81-associated signaling. Sustained IL-6 stimulation increases PKM and LDHA expression in UM cells, thereby enhancing glycolytic flux and lactate production (12). Autocrine lactate subsequently activates GPR81, reduces intracellular cAMP levels and PKA activity, and decreases the ubiquitination and proteasomal degradation of PD-L1 and HLA-E. Increased stability of PD-L1 and HLA-E proteins enhances the resistance of UM cells to cytotoxic T cell- and NK cell-mediated killing. Inhibition of PKM, LDHA, or GPR81 signaling reduces PD-L1 and HLA-E protein levels and enhances immune cell-mediated cytotoxicity. In this context, lactate does not appear to alter the transcription of these two molecules, but instead prolongs their protein stability through receptor-mediated signaling. This study links IL-6-induced glycolytic enhancement, autocrine lactate signaling, and immune evasion in UM, providing a receptor signaling-based perspective for understanding the low immune responsiveness of UM.

Under hypoxic conditions, UM cells may also transfer lactate through extracellular vesicles and influence immune cells. Single-cell transcriptomic analysis revealed that CD63-high tumor cell populations were enriched for gene signatures associated with lactate metabolism and vesicle transport (13). In UMT2 cells, hypoxia increased CD63 expression, extracellular vesicle release, and lactate content within extracellular vesicles. Treatment with hypoxia-derived extracellular vesicles increased CD206 expression and the ECAR/OCR ratio in macrophages, accompanied by elevated IL-10 and TGF-β secretion. CD8^+^ T cells co-cultured with these macrophages exhibited increased PD-1 and TIM-3 co-expression and reduced cytotoxic activity. CD63 silencing attenuated these changes. These findings indicate that, in addition to acting as a soluble metabolite, lactate can also participate in metabolic communication between UM cells and myeloid cells through extracellular vesicle-mediated transport. Because CD63 silencing may simultaneously alter extracellular vesicle abundance and the composition of their associated proteins, nucleic acids, and metabolites, extracellular vesicle-derived lactate is better considered as one component of hypoxic UM-mediated immune microenvironment regulation rather than the sole determinant of immune effects.

Studies in non-UM tumor models suggest that lactylation may also regulate immune cell transcription and immune checkpoint protein stability. In macrophages, lactate accumulation can promote histone lactylation and enhance the transcription of repair-associated genes such as ARG1 (20). In tumor-infiltrating myeloid cells, H3K18la is enriched at the METTL3 promoter region and promotes its expression. Following lactylation at the K281 and K345 sites, METTL3 exhibits enhanced binding capacity toward m^6^A-modified RNAs and subsequently maintains a myeloid immunosuppressive phenotype through YTHDF1-, JAK1-, and STAT3-related signaling. This process involves both promoter-associated histone lactylation and non-histone lactylation of METTL3, indicating that lactylation may sequentially influence transcriptional regulation and RNA recognition functions (43). Other cancer studies have further shown that HAT1-mediated PD-L1 lactylation reduces its endoplasmic reticulum-associated degradation, increases PD-L1 protein stability, and weakens antitumor immune responses (44). In UM, the relevance of these lactylation mechanisms is most likely to depend on the cellular source of lactate, the composition of the myeloid compartment, and the expression of immune checkpoint proteins in primary and metastatic lesions.

Lactate produced by UM cells may participate in immune escape through autocrine receptor signaling and extracellular vesicle-mediated intercellular transfer. Autocrine lactate activates GPR81 signaling and increases the stability of PD-L1 and HLA-E proteins, thereby enhancing tumor cell resistance to T cell- and NK cell-mediated killing. Under hypoxic conditions, lactate may also reach immune cells through extracellular vesicles, driving macrophages toward an immunosuppressive phenotype and reducing CD8^+^ T-cell effector function. By comparison, the role of lactylation in the UM immune microenvironment is still supported mainly by mechanistic studies in other cancer types and myeloid cell models, rather than by direct UM-specific data. For lactylation to be understood in UM immunity, spatial lactate distribution, cell type-specific lactylation patterns, antigen presentation status, and immune cell composition need to be considered together in primary tumors and liver metastases. Selective targeting of lactate metabolism and lactylation may also help separate their relative contributions to myeloid immunosuppression, abnormal antigen presentation, and resistance to immunotherapy.

## 8 Lactate metabolism in the hepatic metastatic microenvironment of UM

Direct data on lactate flux and cell-specific lactylation in UM liver metastases are still limited. This section therefore discusses established features of the UM hepatic niche alongside lactate-related mechanisms reported in other liver metastasis and fibrosis models.

UM spreads mainly through the bloodstream. The liver is the most common metastatic site, and hepatic metastasis remains a major determinant of survival. Within hepatic lobules, oxygen tension, nutrient availability, and metabolic substrates vary by region, creating spatial differences in glycolysis, fatty acid oxidation, gluconeogenesis, and lactate utilization. Liver sinusoidal endothelial cells, Kupffer cells, hepatic stellate cells, and lymphocytes all contribute to the hepatic immune and metabolic microenvironment (45). UM liver metastases usually show limited lymphocyte infiltration and relatively abundant macrophages, often concentrated around tumor regions (46). In a small cohort of patients treated with isolated hepatic perfusion, higher CD68^+^ macrophage infiltration was associated with longer overall survival, whereas CD163^+^ macrophage infiltration showed no similar association (47). Thus, macrophage function in UM liver metastasis cannot be inferred from abundance alone. Their phenotypic characteristics and spatial localization within tumor centers, invasive margins, and adjacent liver tissue also need to be considered. Oxygen and nutrient gradients within hepatic lobules, together with the spatial organization of tumor and host cells, may create regional differences in lactate production, uptake, and oxidative utilization within metastatic lesions.

UM liver metastases show marked heterogeneity in glucose metabolic activity. In a retrospective study of 51 patients, ¹^8^F-FDG uptake varied considerably, even among lesions of similar size. Patients with an SUVmax ≥8.5 had a median overall survival of 9.4 months, whereas those with an SUVmax <8.5 had a median overall survival of 38.4 months (48). These findings suggest that glucose metabolic activity is associated with differences in the biological behavior of hepatic metastatic lesions. ¹^8^F-FDG primarily reflects glucose uptake and phosphorylation but cannot distinguish whether glucose is subsequently utilized for lactate production, mitochondrial oxidation, or biosynthetic pathways. Integrating imaging-based metabolic phenotypes with measurements of lactate levels, LDHA activity, MCT expression, and extracellular acidification status may help determine whether lactate production and utilization differ among distinct hepatic metastatic lesions.

UM cells can modulate hepatic stromal cells through paracrine signaling. VGF secreted by UM cells with GNAQ/GNA11 mutation-associated Gαq/11 signaling activation can stimulate TGF-β-SMAD signaling in hepatic stellate cells through TGFBR2, promoting fibrotic responses and hepatic metastatic colonization (49). BAP1 silencing increases H3K27ac enrichment at the GDF15 promoter and upregulates GDF15, which then induces hepatic stellate cells to produce extracellular matrix components, inflammatory factors, and angiogenic factors (50). Endothelial cells in hepatic metastatic lesions may also enter a KLF4-associated senescent state and, through secreted factors such as CXCL12, promote UM cell migration and local immunosuppression (51). These studies show that hepatic stellate cells and endothelial cells participate in the formation and progression of UM liver metastases. Studies of lactate metabolism in hepatic metastatic lesions should therefore include lactate production, uptake, and transport across several cellular compartments, including UM cells, hepatic stellate cells, and endothelial cells, rather than focusing only on tumor cells.

Studies in other cancers and liver fibrosis offer useful parallels for considering how lactate may act in the hepatic microenvironment. In colorectal cancer liver metastasis models, lactate accumulation and local acidification induce mitochondrial dysfunction and apoptosis in liver-resident NK cells, reducing intrahepatic immune surveillance (52). In liver fibrosis models, HK2-dependent glycolysis and lactate production increase H3K18la and activate hepatic stellate cells. Blocking HK2 or lactate production lowers H3K18la levels and fibrosis-associated gene expression, whereas exogenous lactate partially restores these changes (53). These findings point to two different forms of lactate-associated regulation: suppression of liver-resident NK cells by lactate itself and H3K18la-associated activation of hepatic stellate cells. Whether similar processes occur in UM liver metastases remains unclear and will require analysis of lactate exposure, metabolic changes, and H3K18la levels in NK cells and hepatic stellate cells.

Spatial metabolic analysis combined with cell-type-resolved lactylation profiling could help define which compartments produce or consume lactate in UM liver metastases. Such work would also show whether lactate production, lactate uptake, and H3K18la distribution are linked to impaired NK cell function, hepatic stellate cell activation, vascular remodeling, and metastatic growth.

## 9 Therapeutic implications of targeting lactate metabolism and lactylation

Lactate-associated interventions can target lactate production, local accumulation, transmembrane transport, receptor signaling, and lactylation regulation. These approaches are at different stages of development. Among the lactate transport inhibitors discussed, AZD3965 has entered phase I clinical evaluation in patients with advanced solid tumors or lymphoma, but it has not been evaluated in UM (56). CoMnFe nanozyme-mediated lactate depletion has been tested preclinically in UM, whereas AZD0095 has undergone preclinical evaluation outside UM (55, 57). GPR81 inhibition and direct modulation of lactylation have not undergone therapeutic testing in UM. No lactate- or lactylation-directed treatment discussed in this review has established clinical efficacy in UM. The effects of each approach depend on the responding cell type, local lactate concentration, transport mechanism, and microenvironmental setting.

Inhibiting glycolysis can reduce the pool of pyruvate available for LDHA-mediated lactate production. RASD2 silencing lowers the expression of GLUT1, HK2, PKM2, and LDHA and suppresses glycolytic activity, UM cell growth, and metastasis-associated phenotypes (26). SOX10-regulated GAPDHS also contributes to glucose metabolism in UM, and its silencing reduces glycolytic activity and inhibits xenograft tumor growth (54). These studies indicate that UM cells may depend on specific glycolytic programs. RASD2 and GAPDHS affect more than lactate production, including glucose uptake, energy metabolism, redox homeostasis, and regulation of cellular phenotypes. Assessment of interventions targeting HK2, PKM2, or LDHA should include lactate production, LDHA activity, mitochondrial respiration, and fatty acid oxidation. Their antitumor effects cannot be attributed only to reduced lactate levels.

Local lactate depletion has produced radiosensitizing effects in UM models. A CoMnFe layered double oxide nanozyme converts lactate to pyruvate while catalyzing hydrogen peroxide into oxygen and reactive oxygen species, which can relieve hypoxia and increase radiation-induced tumor damage (55). This approach fits the treatment setting of primary UM, where local therapies remain central. Its antitumor activity reflects several processes, including reduced lactate, improved oxygen availability, increased reactive oxygen species, and stronger radiotherapeutic effects. For this reason, therapeutic assessment should not be limited to lactate levels. Intraocular distribution, biodegradation, long-term retinal toxicity, and tissue responses after repeated administration also need to be considered.

Monocarboxylate transporters control lactate exchange across the cell membrane between tumor cells and neighboring cells. MCT1 can mediate lactate uptake or export, depending on transmembrane lactate and proton gradients. MCT4 mainly supports lactate efflux from highly glycolytic cells. The MCT1 inhibitor AZD3965 has been tested in a phase I dose-escalation study of patients with advanced solid tumors and lymphoma, where it showed measurable pharmacokinetic properties and target engagement (56). The main dose-limiting toxicity in that study was reversible electroretinographic abnormalities. Elevated cardiac troponin and acidosis occurred in individual cases. The electroretinographic toxicity observed with AZD3965 is particularly relevant to ocular applications and indicates the need to monitor retinal structure and visual function.

Because MCT4 can maintain lactate efflux when MCT1 is inhibited, clinical studies have used high MCT1 expression together with low MCT4 expression as an important criterion for patient selection. The selective MCT4 inhibitor AZD0095 has favorable oral pharmacokinetic properties and antitumor activity in animal studies (57). Its efficacy has not been evaluated in UM.

MCT inhibition can affect tumor cells and immune cells at the same time. Activated T cells require MCT1-mediated lactate export to maintain glycolytic and cytotoxic function (39). In UM cells, MCT1 also participates in the uptake of exogenous lactate associated with quiescence-like changes. The effect of an MCT inhibitor will therefore depend on transporter expression and the direction of lactate transport in each cell population. Bulk tissue expression cannot resolve these cell-specific transport functions.

A major challenge for lactylation-targeted therapy is molecular selectivity. In ocular melanoma, p300 intervention has been reported to reduce H3K18la levels and YTHDF2 expression (14). Biochemical studies have shown that HDAC1–3 have histone lysine delactylase activity (23). p300 and HDACs, however, also regulate histone acetylation, other lysine acylation marks, and many non-histone substrates. As a result, antitumor effects from p300 or HDAC inhibition cannot be easily assigned to changes at a single lactylation site. HDAC inhibition may also increase certain lactylation marks while suppressing tumor growth.

UM sensitivity to HDAC and BET inhibitors indicates that epigenetic therapy can alter UM cell states, but it does not show that these effects are mediated specifically by reduced lactylation (58). YTHDF2 and ALKBH3 can be used as readouts of H3K18la-associated transcriptional responses. Targeting these downstream molecules, however, is not equivalent to selectively modulating H3K18la. Therapeutic studies of lactylation will require clearer definition of modification sites, functional substrates, and site-dependent biological effects.

CoMnFe nanozyme-mediated lactate depletion combined with radiotherapy has been tested directly in UM models (55). Autocrine lactate stabilizes PD-L1 and HLA-E in UM cells, but lactate reduction or GPR81 inhibition has not been evaluated in combination with T cell- or NK cell-directed therapy in UM (12). In non-UM models, ALKBH5 deficiency reduces MCT4 expression and tumor interstitial lactate, decreases Treg and MDSC accumulation, and improves the response to anti-PD-1 therapy (59). A comparable response has not been examined in UM.

Lactate-related interventions are currently at different stages of development. Lactate depletion combined with radiotherapy has been tested in UM models (55), whereas MCT1 and MCT4 inhibitors have undergone pharmacological or clinical evaluation in other cancers (56, 57). GPR81 inhibition and direct modulation of lactylation have not yet been tested therapeutically in UM. **Table 1** presents these approaches alongside their molecular mechanisms, study systems, and current limitations.

**Table 1:** Lactate-associated mechanisms, therapeutic strategies, and development status in UM and related studies.

Process	Mechanism and main observations	Study system	Potential agents or strategies	Main considerations	References
Intraocular lactate accumulation and local lactate depletion	L-lactate is elevated in the aqueous humor of patients with UM and is associated with pathological mitotic activity	Aqueous humor from patients with UM; UM cell and animal studies	CoMnFe layered double oxide nanozyme combined with radiotherapy [UM preclinical]	Antitumor activity involves lactate depletion, hypoxia relief, and ROS generation; intraocular distribution, biodegradation, and retinal toxicity require evaluation	(10, 55)
Glycolysis-associated lactate production	HK2-, RASD2-, and GAPDHS-related glycolytic programs regulate glucose metabolism, lactate production, and malignant phenotypes in UM	UM cells and xenograft studies	Modulation of RASD2 or GAPDHS [UM preclinical]; direct inhibition of HK2, PKM2, or LDHA [not therapeutically tested in UM]	These targets also affect glucose uptake, energy metabolism, and redox balance, so treatment effects cannot be attributed solely to reduced lactate production	(25, 26, 54)
Metabolic reprogramming in high-risk UM	BAP1-mutant UM shows variable dependence on glycolysis, nucleotide synthesis, fatty acid oxidation, and altered pyruvate metabolism	BAP1-mutant UM samples and cell studies	Selection of metabolic interventions according to glycolytic or oxidative phenotype [not therapeutically tested]	Metabolic heterogeneity may produce different responses to lactate-directed treatment	(31, 32)
MCT-mediated lactate transport and quiescence-like adaptation	MCT1 participates in lactate uptake associated with a low-proliferative state, whereas MCT1 and MCT4 regulate lactate exchange across tumor and immune cells	UM cells; pharmacological studies in other cancers	AZD3965 [Phase I, non-UM]; AZD0095 [preclinical, non-UM]; other MCT1- or MCT4-targeted approaches [not therapeutically tested in UM]	MCT inhibition may affect UM cells, effector T cells, and retinal metabolism; MCT4 may compensate for MCT1 inhibition	(11, 39, 56, 57)
HCAR1/GPR81-mediated immune regulation	Autocrine lactate stabilizes PD-L1 and HLA-E through GPR81 signaling and increases resistance to T cell- and NK cell-mediated cytotoxicity	UM cells and immune functional assays	Reduction of lactate production or HCAR1/GPR81 inhibition [mechanistic; not therapeutically tested in UM]; combination with immune checkpoint blockade or T cell-directed therapy [not therapeutically tested in UM]	UM-specific *in vivo* therapeutic studies and clinical data are limited	(12)
Extracellular vesicle-associated metabolic communication	Hypoxia-associated extracellular vesicles carry lactate to immune cells, alter macrophage states, and reduce CD8^+^ T-cell effector function	Hypoxic UM cells and immune-cell assays	Interference with extracellular vesicle production, lactate loading, release, or uptake [mechanistic; not therapeutically tested in UM]	The relevant extracellular vesicle targets and their effects *in vivo* have not been established in UM	(13)
Histone and non-histone lactylation	Lactylation affects chromatin regulation, protein stability, enzymatic activity, localization, and molecular interactions	General tumor and cellular studies	Modulation of p300, ACSS2-KAT2A, HDAC1–3, SIRT1, or SIRT3 [mechanistic; no lactylation-specific clinical validation]	These enzymes also regulate acetylation and other lysine acylation modifications, limiting lactylation-site selectivity	(20, 22–24)
H3K18la-associated m^6^A regulation	Promoter-associated H3K18la increases YTHDF2 expression and promotes degradation of m^6^A-modified PER1 and TP53 transcripts	Ocular melanoma tissues and UM-derived and conjunctival melanoma cell lines	Modulation of H3K18la-related enzymes or YTHDF2 [mechanistic; ocular melanoma studies]	Targeting YTHDF2 does not selectively regulate H3K18la; the mechanism has not been characterized separately across UM subtypes and liver metastases	(14)
H3K18la-associated m¹A regulation	H3K18la increases ALKBH3 expression and alters m¹A-dependent stability and translation of SP100A-related transcripts	Ocular melanoma tissues and UM-derived and conjunctival melanoma cell lines	ALKBH3 inhibition or modulation of H3K18la-related enzymes [mechanistic; ocular melanoma studies]	ALKBH3 acts on multiple RNA substrates, and the reported experiments included ocular melanoma cells of different origins	(15)
RNA modification networks	YTHDF1, ALKBH5, METTL3, and related factors regulate transcript stability and translation in UM and ocular melanoma	UM and ocular melanoma samples, cell studies, and non-UM immune studies	Modulation of YTHDF1, ALKBH5, METTL3, or related RNA regulators [mechanistic]; ALKBH5-related combination with anti-PD-1 therapy [preclinical, non-UM]	These approaches have broad post-transcriptional effects and do not selectively target lactylation	(33–35, 59)

Development status refers to the disease setting in which each strategy has been evaluated. “UM preclinical” indicates evaluation in UM cell or animal studies; “preclinical, non-UM” indicates evaluation outside UM; “Phase I, non-UM” indicates clinical evaluation in patients with cancers other than UM; and “mechanistic” indicates pathway or functional studies without therapeutic testing. None of the listed lactate- or lactylation-directed strategies has established clinical efficacy in UM.

## 10 Discussion

The studies reviewed here follow the progression from lactate production and transport to cellular uptake, receptor signaling, extracellular vesicle-associated transfer, and lactylation. In UM, lactate uptake has been associated with a low-proliferative state, whereas GPR81 signaling and extracellular vesicle-associated lactate transfer affect tumor immune resistance and immune-cell function. Lactylation may add chromatin and RNA regulatory effects, although its UM-specific roles remain less well defined. The central question is how these different modes of lactate action contribute to cell-state regulation, immune escape, and hepatic metastatic adaptation. Lactylation is therefore discussed as one part of lactate-related regulation, not as an established central mechanism of immunotherapy response in UM.

Lactate-induced low-proliferative states may be relevant to delayed metastasis in UM, although they are not equivalent to tumor dormancy *in vivo* (11). Reduced proliferation does not necessarily indicate diminished metastatic potential if cells remain viable and retain the ability to re-enter the cell cycle. The concurrent increase in H3K18la may reflect chromatin changes associated with this state. Cell-cycle re-entry and clonogenic regrowth after lactate withdrawal are needed to determine whether the phenotype is reversible.

H3K18la should not be assigned a fixed oncogenic or tumor-suppressive role. In the Longhitano study, increased H3K18la accompanied a low-proliferative state with enhanced oxidative metabolism. By contrast, ocular melanoma studies linked promoter-associated H3K18la to YTHDF2 or ALKBH3 expression and tumor-promoting RNA regulation (14). This difference may arise from tumor origin, lactate exposure, metabolic state, and the genomic distribution of H3K18la. Total H3K18la abundance alone cannot indicate which genes are being regulated or whether the resulting cellular state favors persistence or proliferation. Current ocular melanoma studies have focused mainly on histone H3K18la, while the effects of non-histone lactylation in UM remain largely undefined.

A pan-cancer multi-omics analysis reported that the prognostic association of a transcriptome-derived lactylation score varied among tumor types. In UVM, a higher lactylation score was associated with favorable prognosis. Across the pan-cancer cohort, higher scores were associated with lower infiltration of CD8^+^ T cells, γδ T cells, NKT cells, and NK cells and with poorer immunotherapy response (60). The UVM result differs from the reported association between elevated H3K18la and poor clinical outcome in ocular melanoma. These measurements are not equivalent: the lactylation score was calculated from the expression of lactylation-related genes, whereas H3K18la represents a specific histone modification.

H3K18la-associated regulation of YTHDF2 and ALKBH3 in ocular melanoma illustrates how lactylation-related transcriptional changes can affect RNA degradation, stability, and translation (14, 15). In these studies, altered YTHDF2 or ALKBH3 expression affected specific downstream transcripts. Studies of YTHDF1-HINT2, ALKBH5-FOXM1, and m^6^A regulator expression in UM also associate RNA regulation with tumor phenotypes and prognosis. Whether H3K18la contributes to these processes in UM remains unclear.

In the context of immune escape, lactate can act on both UM cells and surrounding immune cells. IL-6-induced autocrine lactate promotes PD-L1 and HLA-E protein stability through GPR81 signaling, thereby enhancing the resistance of UM cells to T cell- and NK cell-mediated cytotoxicity (12). Hypoxia-derived extracellular vesicles carrying lactate can influence macrophage and CD8^+^ T cell functions, suggesting that lactate may also mediate metabolic communication between tumor cells and immune cells (13). These processes may represent metabolic immune regulatory mechanisms in addition to low tumor mutational burden, ocular immune privilege, abnormal antigen presentation, and hepatic immune tolerance.

H3K18la in myeloid cells, METTL3 lactylation, and PD-L1 lactylation have mainly been reported in other tumor types. Their cellular distribution and functional effects in primary UM, liver metastases, and coculture studies remain unclear. Distinguishing the effects of lactate metabolism, GPR81 receptor signaling, and lactylation modifications will help clarify the relative contributions of different lactate-associated processes to immune hyporesponsiveness in UM.

Glucose metabolic heterogeneity in UM liver metastases complicates the assessment of local lactate metabolism. FDG uptake does not distinguish glucose use for lactate production from mitochondrial oxidation or biosynthesis. Hepatic stellate cells, endothelial cells, and resident immune cells may also contribute to local lactate production and utilization. Studies in other liver metastasis and fibrosis settings describe lactate-associated impairment of liver-resident NK cells and H3K18la-associated hepatic stellate cell activation (52, 53). Whether these processes occur in UM liver metastases requires examining lactate production, transport, and utilization in the relevant cell populations.

Lactate metabolic interventions and direct targeting of lactylation are at different stages of development. Approaches targeting lactate production, local accumulation, or transport have undergone experimental or pharmacological evaluation, but their effects also involve energy metabolism, redox balance, intracellular pH, and immune cell function. In UM, therapeutic assessment should distinguish changes in tumor cell lactate export and uptake from effects on immune cells and retinal function. Direct targeting of lactylation is limited by the lack of selectivity of available agents. Enzymes that regulate lactylation also act on acetylation and other lysine acylation modifications, making it difficult to attribute the effects of their inhibition to individual lactylation sites (22–24). Identifying the relevant substrate proteins and modification sites is therefore necessary to determine how lactylation contributes to treatment response.

Changes in pan-Kla or H3K18la abundance after treatment do not show whether a particular lactylation site mediates the treatment response. Measurements of lactylation abundance and chromatin distribution should be paired with analyses of target-gene expression, substrate function, immune-cell activity, and metastatic phenotypes.

Lactate has been studied directly in UM in relation to low-proliferative states and immune resistance. The roles of lactylation in RNA regulation and of lactate in liver metastases are less well defined. Comparisons between primary UM and liver metastases may clarify whether these processes change during metastatic progression and influence treatment response.

## 11 Conclusion

In UM, lactate-related regulation includes changes in lactate production and transport, effects mediated through HCAR1/GPR81 and extracellular vesicles, and lactylation-associated chromatin and RNA regulation. Current studies indicate that lactate may contribute to low-proliferative tumor cell states, PD-L1 and HLA-E protein stability, immune cell dysfunction, and metabolic communication between tumor and immune cells. These effects vary with lactate source, local concentration, transport mode, and the metabolic state of recipient cells. Lactylation may further extend lactate-associated metabolic changes to chromatin regulation and RNA modification, with H3K18la-mediated regulation of YTHDF2 and ALKBH3 representing one of the more clearly described mechanisms in ocular melanoma studies. Future work should clarify how lactate production and transport, lactylation sites, RNA modifications, immune function, and metastatic phenotypes are related in UM clinical samples, patient-derived models, and liver metastases. This will help define the biological significance and translational potential of lactate metabolism and lactylation in delayed metastasis, limited responsiveness to immune checkpoint blockade, and combination therapy in UM.
